# Fyn Kinase Is Required for Optimal Humoral Responses

**DOI:** 10.1371/journal.pone.0060640

**Published:** 2013-04-08

**Authors:** Natalia S. Chaimowitz, Yves T. Falanga, John J. Ryan, Daniel H. Conrad

**Affiliations:** 1 Department of Microbiology and Immunology, Virginia Commonwealth University School of Medicine, Richmond, Virginia, United States of America; 2 Department of Biology, Virginia Commonwealth University, Richmond, Virginia, United States of America; McGill University, Canada

## Abstract

The generation of antigen-specific antibodies and the development of immunological memory require collaboration between B and T cells. T cell-secreted IL-4 is important for B cell survival, isotype switch to IgG1 and IgE, affinity maturation, and the development of germinal centers (GC). Fyn, a member of the Src family tyrosine kinase, is widely expressed in many cell types, including lymphocytes. This kinase is known to interact with both the B cell and T cell receptor (BCR and TCR, respectively). While Fyn deletion does not impair the development of immature T cells and B cells, TCR signaling is altered in mature T cells. The current study demonstrates that Fyn deficient (KO) B cells have impaired IL-4 signaling. Fyn KO mice displayed low basal levels of IgG1, IgE and IgG2c, and delayed antigen-specific IgG1 and IgG2b production, with a dramatic decrease in antigen-specific IgG2c following immunization with a T-dependent antigen. Defects in antibody production correlated with significantly reduced numbers of GC B cells, follicular T helper cells (T_FH_), and splenic plasma cells (PC). Taken together, our data demonstrate that Fyn kinase is required for optimal humoral responses.

## Introduction

The development of immunological memory is critical for long-term protection against pathogen infection. GCs are structures required for the development of a proper humoral immunity. Within GCs, B cells undergo class switch recombination (CSR) and somatic hypermutation (SHM), leading to the development of PCs that secrete high affinity, class switched antibodies. GC formation is dependent on the interactions between antigen-specific B cells, T cells and follicular dendritic cells (FDCs). During B cell-T cell interaction, T_FH_ provide B cell help via CD40L and cytokines, such as IL-21 and IL-4. Both of these cytokines are important for germinal center formation. Even prior to the discovery of T_FH_ cells, it was demonstrated that optimal GCs did not form in IL-4Rα or IL-4 KO mice, suggesting the importance of this cytokine pathway. In addition to being required for GC formation, IL-4 signaling pathway is also critical for immunoglobulin class switch recombination and somatic hypermutation [1]–[3].

Cytokine receptor signaling comprises a diverse array of intracellular molecules including kinases and phosphatases. Here we focused on Fyn kinase, a member of the Src protein tyrosine kinase family, widely expressed in many cell types such as lymphocytes [1]–[4]. Fyn interacts with SLAM-associated protein (SAP) [5]–[7], generating a ternary complex with SLAM, which leads to SLAM tyrosine phosphorylation. This interaction generates docking sites for several proteins and initiates signaling cascades [8], [9]. Fyn has been shown to interact with both the B cell and T cell receptor (BCR and TCR, respectively) [10], [11]. While Fyn deletion did not impair the development of immature T cells and B cells, TCR signaling was altered in mature T cells [1]–[3], [12], [13].

A role for Fyn in antibody production has seemed logical, given that its loss reduces both T and B cell proliferation [4], [14]–[16]. Fyn interacts with both the BCR and the BCR co-receptor, CD19 [5]–[7], [17]. Surprisingly, Fyn KO B cells showed only mildly impaired BCR signaling [8], [9], [18]. Moreover, humoral responses to T-dependent antigens were not statistically different in Fyn KO mice 7 or 30 days post-immunization [10], [11], [18], [19]. On the other hand, Fyn-deficient (KO) mice displayed a marked decrease in Ig subsequent to T-independent antigens, suggesting a role for Fyn in this immunization protocol [13], [20]. Experiments with Fyn-KO mice demonstrated that they were able to form GCs, although the absolute number of cells comprising these was not assessed [19].

In addition to its role in T-independent antigen responses, Appleby and co-workers showed that Fyn was required for B cell responses to IL-5 [18]. With the current understanding of T_FH_ function, and a renewed importance for IL-4, we re-evaluated the role of Fyn in humoral responses, attempting to isolate the role of this kinase to the B cell. Our results demonstrate that Fyn KO B cells have decreased antibody production following *in vitro* activation (anti-CD40 and IL-4), coincident with impaired STAT3 and STAT5 phosphorylation upon IL-4Rα stimulation. Moreover, Fyn KO mice not only had significantly lower basal levels IgE, IgG1 and IgG2c, but also showed impaired antibody production *in vivo* upon immunization. The impairment was characterized by a delay in the production of antigen-specific IgG1 and IgG2b, and significantly reduced IgG2c. The defects in antibody production correlated with reduced numbers of GC B cells, T_FH_ cells and splenic PCs. Our results thus demonstrate that Fyn-mediated signaling in B cells is necessary for optimal humoral responses.

## Materials and Methods

### Animals and Immunizations

C57BL/6x129sv wild type (WT) and C57BL/6x129 sv Fyn-deficient (KO) inbred strains were described previously [21], [22]. Of note, WT and Fyn-deficient KO mice were background matched. All mice were used at a minimum of 9 weeks of age, and all experiments received approval from the Virginia Commonwealth University Institutional Animal Care and Use Committee (IACUC). Immunizations were comprised of injections of 10 µg 4-Hydroxy-3-nitrophenyl-acetyl coupled to keyhole limpet hemocyanin at a ratio of 27∶1 (NP_27_KLH, referred to as NP-KLH) in 4 mg of alum or 100 µg of NP-LPS in PBS (Biosearch Technologies).

### In vitro Activation of Antibody Production

Naïve B cells were isolated using magnetic beads following the manufacturer’s protocol (Miltenyi Biotec). Briefly, a single cell suspension was generated and cells were resuspended in MACS buffer (PBS pH 7.2, 0.5% BSA and 2 mM EDTA) and incubated with anti-CD43 magnetic beads for 15 minutes at 4°C. Cells were then washed and passed through a magnetic column. Flow-through was collected. For IgG1 and IgE production, B cells were cultured with 1 µg/mL of stimulating anti-CD40 antibody (Invitrogen) and IL-4 (10,000 U/mL) for seven days. Supernatants were then harvested and analyzed for IgG1 and IgE production by ELISA. For western blots, B cells were stimulated with 30 ng of IL-4 (Peprotech).

### ELISA

For total IgM and IgG1 ELISAs, samples were serially diluted and added to 96-well plates (50 µL/well) pre-coated with 5 µg/mL of goat-anti IgM and IgG, respectively (Southern Biotech). For standard curve, normal mouse IgM and IgG1 (Southern Biotech) were used. After incubation at 37°C for 1 h, bound Abs were revealed by goat-anti-IgM-alkaline phosphatase (AP) and goat-anti-IgG1-AP, respectively (Southern Biotech). Total IgE ELISA was carried out as previously described [23]. For antigen-specific ELISAs, ELISA was carried out as described with minor modifications [24]. Plates were coated with NP_14_BSA (Biosearch Technologies)(15 µg/mL in PBS) for samples and with 5 µg/mL of goat-anti Ig (Southern Biotech) in BBS for standard. Standard curves were performed by coating with anti-IgM, anti-IgG1, anti-IgG2a or IgG2b and adding known amounts of IgM, IgG1, IgG2a or IgG2b, respectively. The values for experimental samples are reflected as relative units (RU) because serum antigen-specific Abs were captured with antigen, while standard was captured using an anti-IgG specific to the Ig subset of interests. The remaining steps were carried out as described.

### Proliferation

To determine proliferation, cells were isolated and stimulated as described (see above). After 96 hours in culture, cells were pulsed with 1 µCi/well of [^3^H]-thymidine (Perkin Elmer) for 24 hrs. Plates were then harvested onto GFC plates using a filtermate cell harvester. Plates were dried for at least 2 hours, 25 µL of scintillation fluid was added, and incorporated thymidine determined using the Topcount Plate Counter (Perkin Elmer, Waltham, MA).

### Flow Cytometry

Cell isolation and labeling was conducted as described previously [24]. Cells were labeled following erythrocyte lysis and filtration through 40 µm cell strainers. Abs included anti-mouse unlabeled 2.4G2 for Fc block, biotinylated CXCR5 (RF8B2); PE-conjugated Fas (Jo2); APC-conjugated GL7 (GL7), PE-Cy7 conjugated-streptavidin from BD Bioscience; FITC-conjugated B220 (RA3-6B2), CD4 (RM4-5), and PerCP-Cy5.5 conjugated PD-1 (29F.1A12) from Biolegend. Lymphocytes were gated based on FSC vs SSC. Cell subsets were defined as follows: Plasma cells: B220^lo/−^CD138^+^ [25]; germinal center B cells: B220^+^GL7^+^Fas^hi^ [26] and follicular helper T cells: CD4^+^CXCR5^+^PD-1^hi^ [27]. Flow cytometric analysis was performed using a Canto or Aria II (BD Biosciences), and data analysis was conducted with FlowJo (Tree Star).

### Western Blot

Cells were dissociated in RIPA (Radio-Immunoprecipitation Assay) buffer and Western blotting was performed using 50 µg total cell lysate per sample. Proteins were loaded and separated over an 8–16% gradient SDS-polyacrylamide gel (Bio-Rad, Hercules, CA). Proteins were transferred to nitrocellulose membranes (Pall Corporation, Ann Arbor, MI), and blocked for 60 minutes in Blotto B buffer (Rockland Immunochemicals, Gilbertsville, PA) plus 0.1% Tween-20. Blots were incubated in a solution of TBS supplemented with 0.1% Tween-20 and 5% BSA (TBST), with the indicated antibodies overnight at 4°C with gentle rocking. Blots were washed six times for 5 minutes each in TBST, followed by incubation in Blotto B containing a 1∶5,000 dilution of HRP linked anti-IgG matched to the relevant species, from Cell Signaling (Danvers, MA). Size estimates for proteins were obtained using molecular weight standards from Bio-Rad (Hercules, CA).

### Statistical Analysis

Data are presented as the mean plus or minus SEM of at least 3 independent experiments. Comparisons were made by the two-tailed Student t-test. P<0.05 values were considered statistically significant. *p<0.05, **p<0.01, ***p<0.001. Statistical analysis was performed with GraphPad Prism software.

## Results

### Fyn Kinase Deficiency Leads to Impaired Antibody Production in vivo

**Figure 1:**
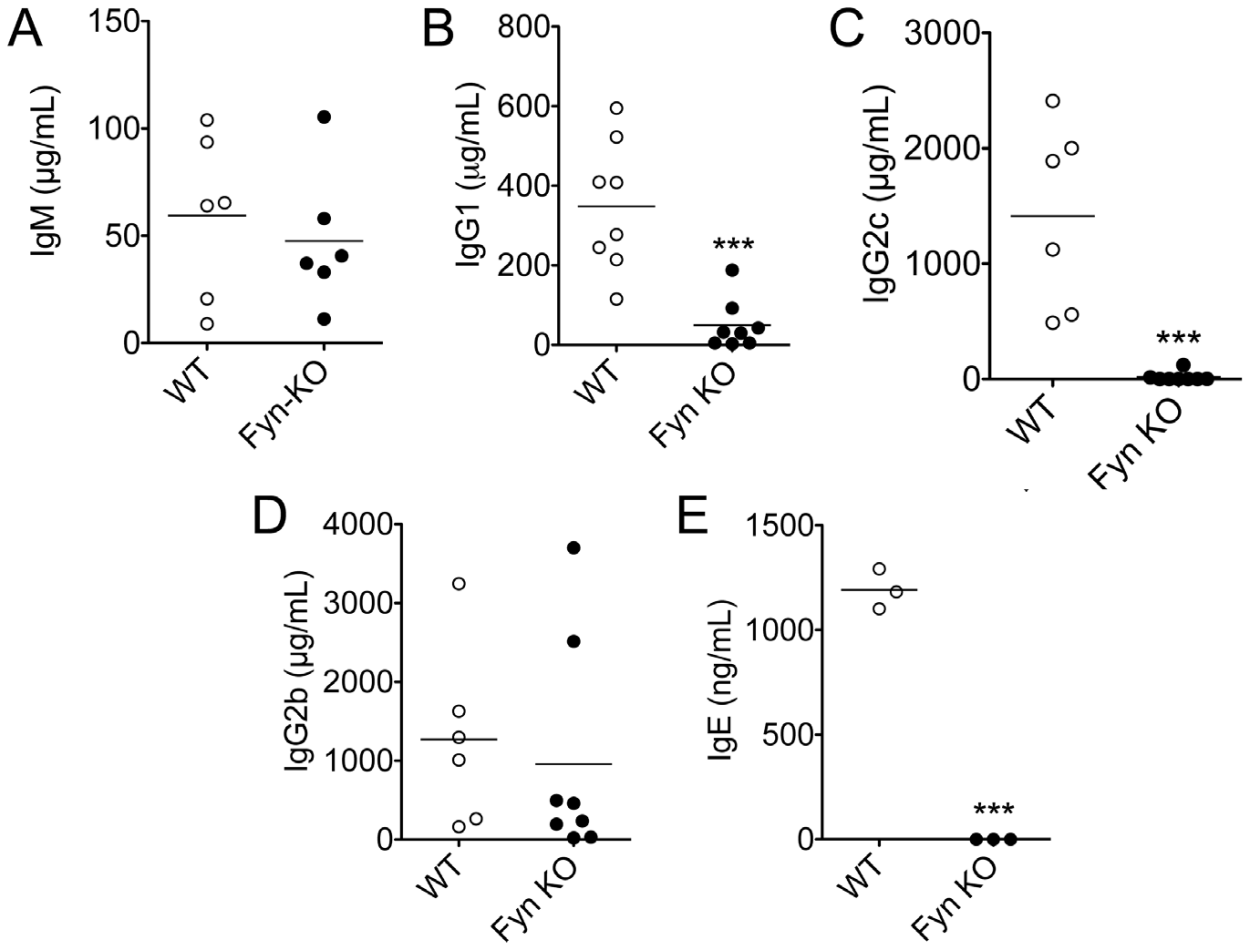
Fyn deficient mice have impaired humoral responses. Serum (a) IgM, (b) IgG1, (c) IgG2c, (d) IgG2b and (d) IgE were measured by ELISA from 8–12 week old naïve mice. Bars represent the mean ± SE of 10 mice per group (***p<0.001). Data represent results obtained in at least two independent experiments.

Circulating antibodies play a key role in immune mechanisms such as pathogen neutralization, opsonization and cell activation. Consequently, we sought to determine whether Fyn plays a role in antibody production *in vivo.* To this end, we analyzed antibody levels in the serum of naïve, age-matched Fyn KO and WT B6.129 mice. Compared to WT, Fyn KO mice had normal IgM and IgG2b levels, but significantly decreased basal IgG1 and, and virtually no IgG2c or IgE (Figure 1).

**Figure 2:**
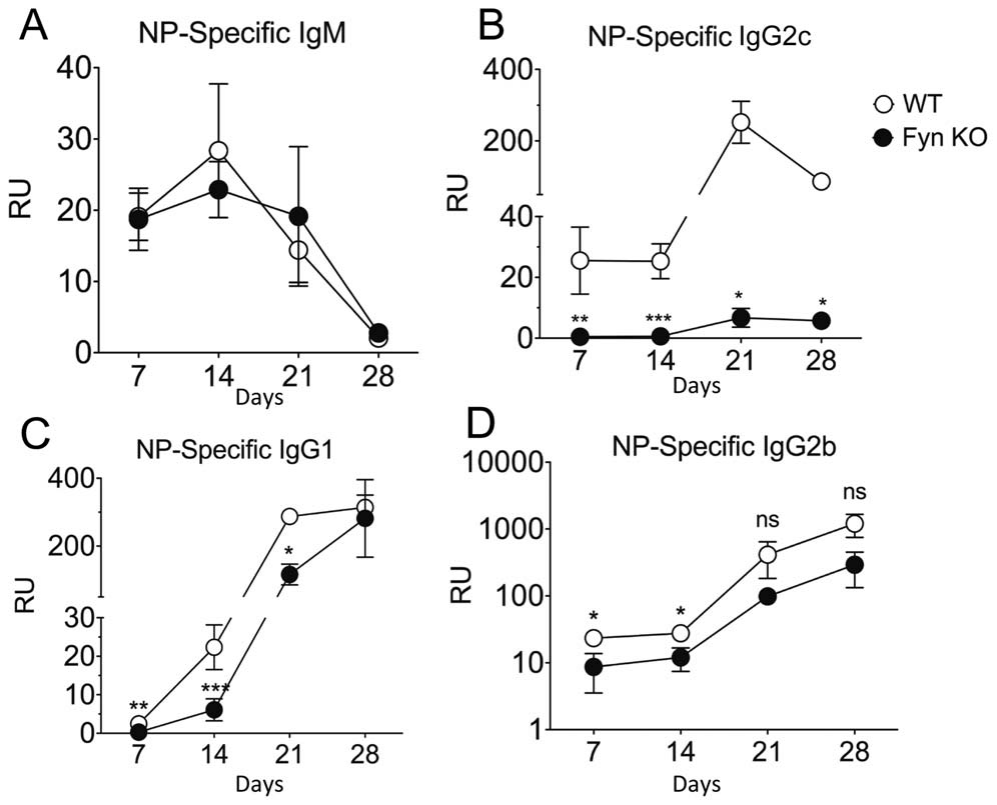
Fyn KO mice have impaired antibody titers upon T-dependent immunization. WT and Fyn KO mice (n  = 5–9) were immunized i.p. with 10 ìg of NP-KLH emulsified in 4 mg of Alum. NP-specific antibody titers were assessed by ELISA: (a) IgM, (b) IgG2c, (c) IgG1 and (d) IgG2b after 7, 14, 21 and 28 days following immunization. Data shown are mean ± SE of 2 independent experiments. *p<0.05; **p<0.001; ***p<0.0001 based on Student’s t test on Fyn WT and Fyn KO. RU, relative units.

To directly assess antigen-specific antibody production, age-matched Fyn KO and WT mice were immunized i.p. with NP-KLH in alum and the generation of NP-specific antibodies was monitored by ELISA. Consistent with our data from naïve mice, IgM levels in Fyn KO and WT mice were similar. Although NP-specific IgG1 was initially lower in Fyn KO mice, its concentration gradually increased to be commensurate with WT levels by 4 weeks post-immunization. A similar trend was also observed for IgG2b titers (Figure 2). The severely low levels of IgG2c we observed in naïve mice were mirrored by very weak induction of IgG2c in immunized Fyn KO mice. Importantly, we observed that NP-specific IgG2c titers in Fyn KO mice were more than 10-fold lower than their WT controls even four weeks after the immunization. These results demonstrate that Fyn has selective roles in isotype switching, with an important role in IgG2c secretion.

### Fyn KO Mice have Reduced Splenic Plasma Cell Numbers

**Figure 3:**
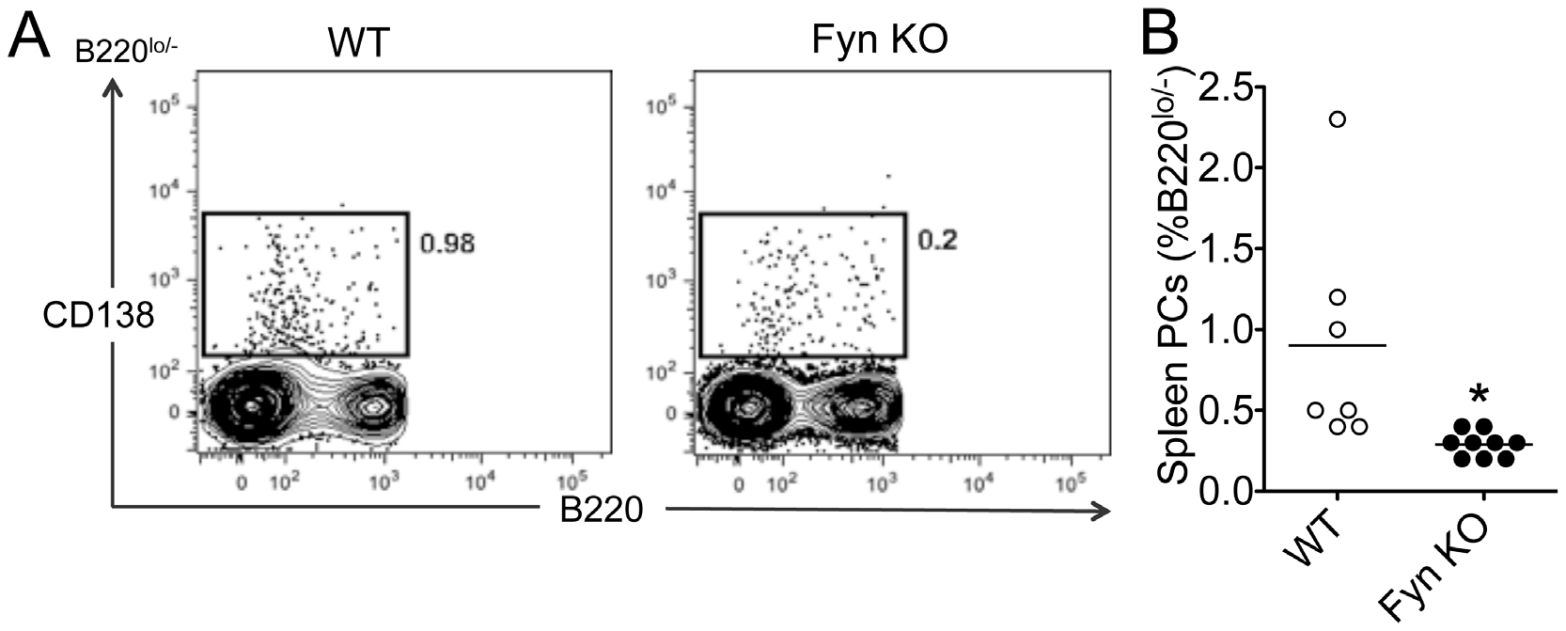
Fyn-KO mice have reduced plasma cell percentages. Fyn-KO and WT mice were immunized with NP-KLH emulsified in alum. Twenty-one days following immunization, spleens were harvested and PC numbers were analyzed. (A) Representative FACS staining. (B) Quantified results from spleen. Bars represent the mean ± SE of 6 mice per group. Data represent results obtained in at least two independent experiments. (**p<0.01).

Plasma cells (PC) are specialized in producing antibodies in both homeostatic and pathological conditions. Given the diminished antibody response observed in Fyn KO mice, we hypothesized that either PC development or their ability to produce antibodies (or both) was impaired in these mice. To verify this hypothesis, we immunized mice with NP-KLH and determined the frequency of PC by flow cytometry 14 days post-immunization. Interestingly, we observed that Fyn KO mice displayed significantly decreased splenic PC (CD138^+^B220^lo/−^), consistent with the defects in antibody production reported earlier (Figure 3).

### Fyn KO Mice have Impaired GC Formation

**Figure 4:**
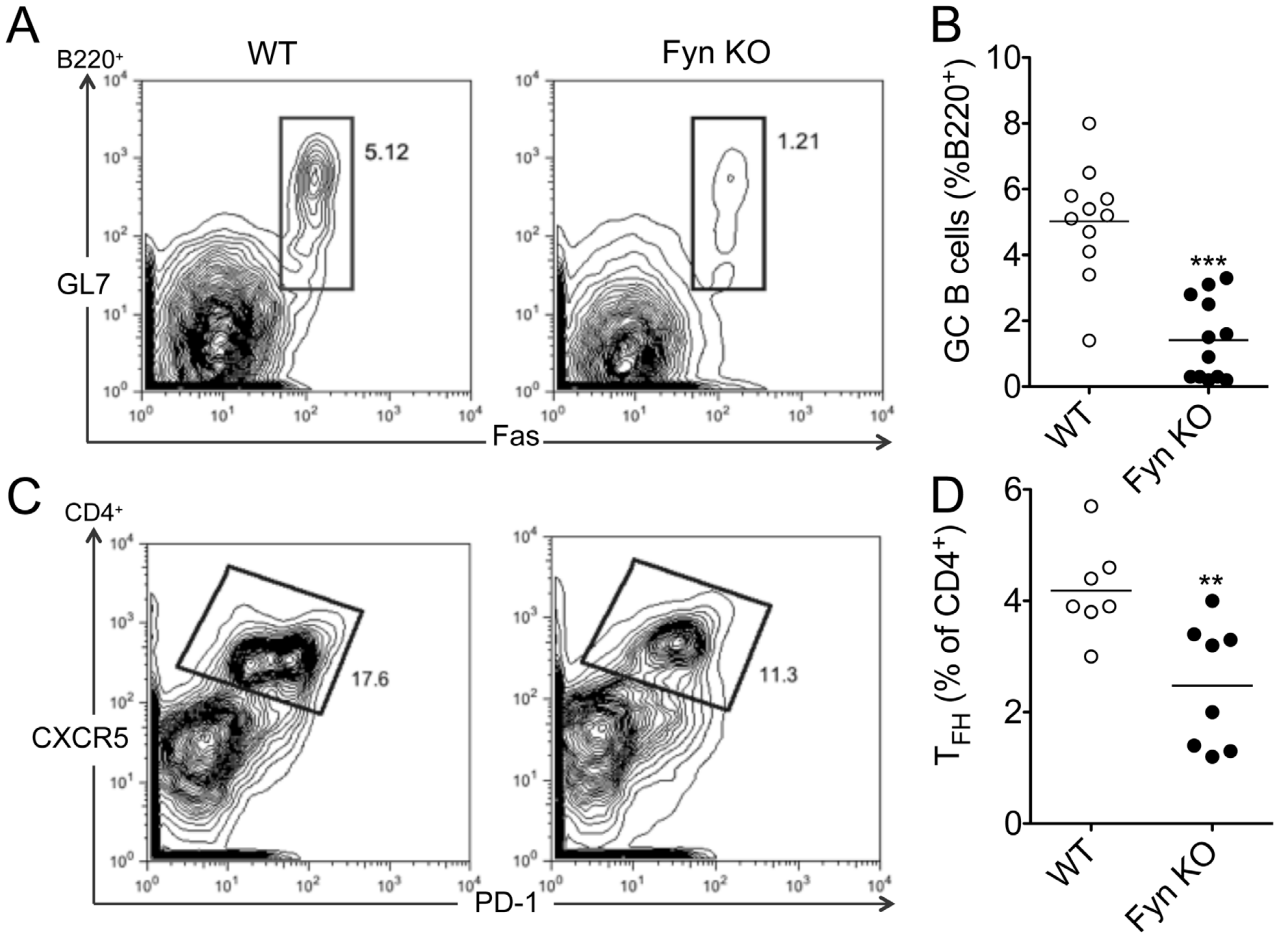
Fyn deficient mice have impaired germinal center formation and reduced T_FH_ numbers. Mice were immunized with NP-KLH emulsified in alum and 14 days post-immunization GC formation and T_FH_ frequency were assessed by flow cytometry. (A) Representative dot plot for GC B cells (gated on B220^+^ cells). (B) Quantification of GC B cells. (C) Representative dot plot for T_FH_ cells (gated on CD4^+^ cells) (D) Quantification of T_FH_ cells. Bars represent the mean ± SE of 6 mice per group. Data represent results obtained in at least two independent experiments. (**p<0.01, ***p<0.001).

A large portion of PC generated in response to T-dependent antigens are derived from GCs. We therefore assessed GC formation in immunized Fyn KO mice and their WT controls by enumerating GC B cells, defined as B220^+^GL7^+^Fas^hi^ 14 days following NP-KLH immunization. Our results demonstrate that 14 days post-immunization, Fyn KO mice displayed significantly decreased levels of GC B cells compared to WT controls (Figures 4a, 4b).

### T_FH_ Formation is Impaired in Fyn Deficient Mice

T_FH_ cells provide B cell help via CD40L, IL-21 and IL-4. They play a key role in GC B cell survival, affinity maturation, and plasma cell differentiation and are characterized by high expression of CXCR5 and programmed death-1 (PD-1). In order to assess whether the decreased number of GC B cells was linked to a defect in the T_FH_ population, we enumerated CD4^+^CXCR5^+^PD1^+^ T_FH_ cells 14 days following NP-KLH immunization. We observed that the percentage of T_FH_ was moderately but consistently reduced in Fyn KO mice and was consequently linked to the noted impaired germinal center formation and low plasma cells (Figures 4c, 4d).

### Fyn Kinase is Required for B Cell Antibody Production in vitro

**Figure 5:**
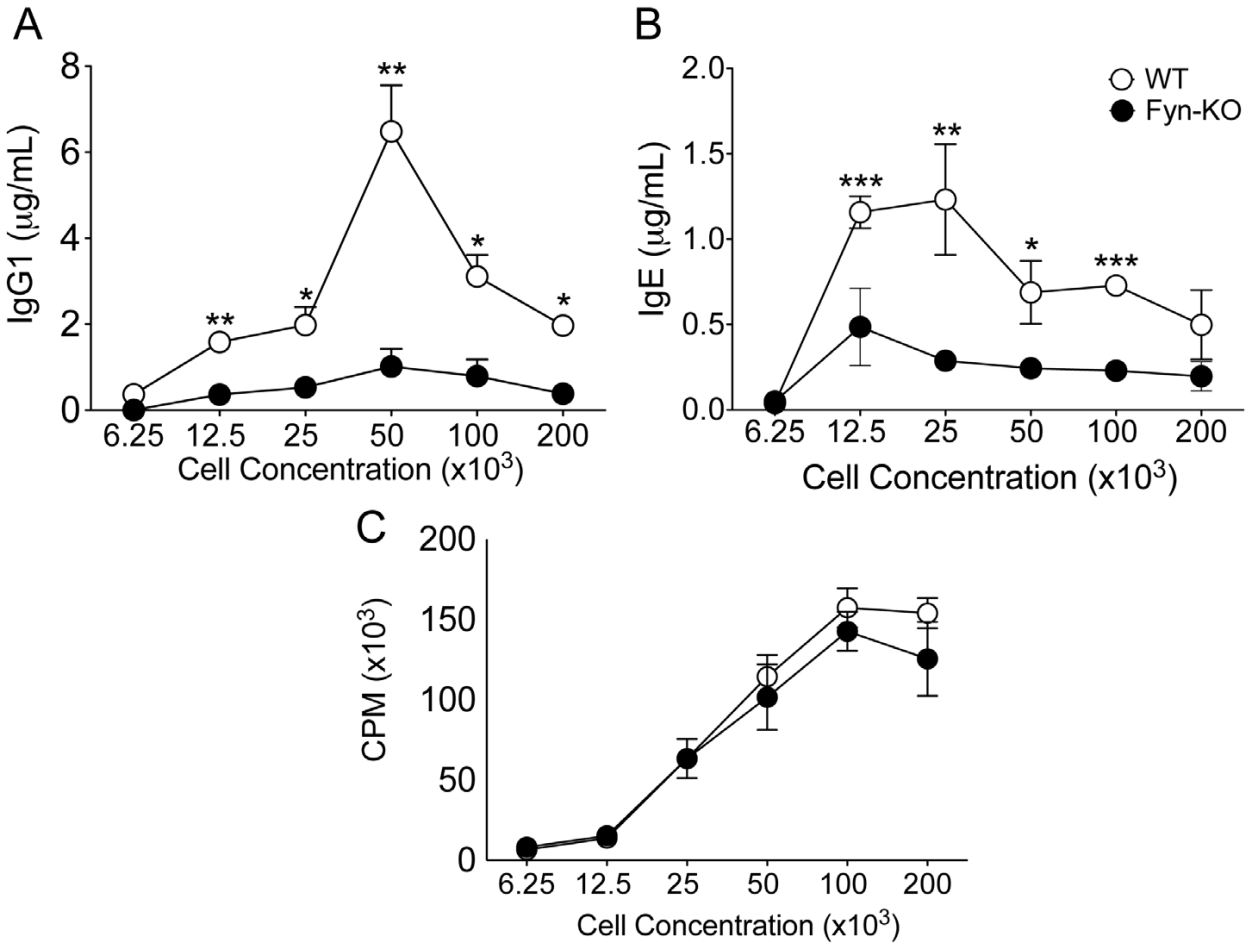
Fyn deficient B cells have reduced antibody levels despite normal proliferation following *in vitro* stimulation. B cells were isolated and cultured with IL-4 and anti-CD40L. Eight days following stimulation, supernatant were harvest and analyzed for (a) IgG1 and (b) IgE by ELISA. Three days following challenge, tritiated thymidine was added to cells. Cells were incubated for 24 hours and then harvested. Thymidine incorporation was then measured (c). Experiment has been performed three times with similar results. (*p<0.05, **p<0.01, ***p<0.001).

T-dependent antigen immunization is a complex process principally involving DCs, T and B cells. While numerous studies investigated the role of Fyn kinase in T cell-mediated antibody production [1], [28]–[30] we sought to determine the contribution of this kinase in B cell survival and antibody production by studying Fyn KO B cells. To this end, WT and Fyn KO B cell were freshly isolated from age-matched mice and stimulated *in vitro* with anti-CD40 and IL-4. Cells were plated at different concentrations as cell concentration can impact antibody production *in vitro.* Interestingly, we observed that Fyn KO B cells produced significantly lower amounts of IgG1 and IgE compared to their WT controls (Figures 5a, 5b). Note that several cell concentrations were examined for these experiments, in view of the finding that in vitro Ig production is highly cell concentration dependent [23], [31]. Furthermore, given that IgE production requires multiple rounds of cell division, we decided to assess whether Fyn deletion also impaired B cell proliferation. We observed that Fyn KO B cells proliferated similarly to their WT controls (Figure 5c), suggesting that Fyn KO B cells have impaired activation-induced isotype switching that is unrelated to proliferation *in vitro*.

### Fyn KO B Cells have Impaired IL-4 Signaling

**Figure 6:**
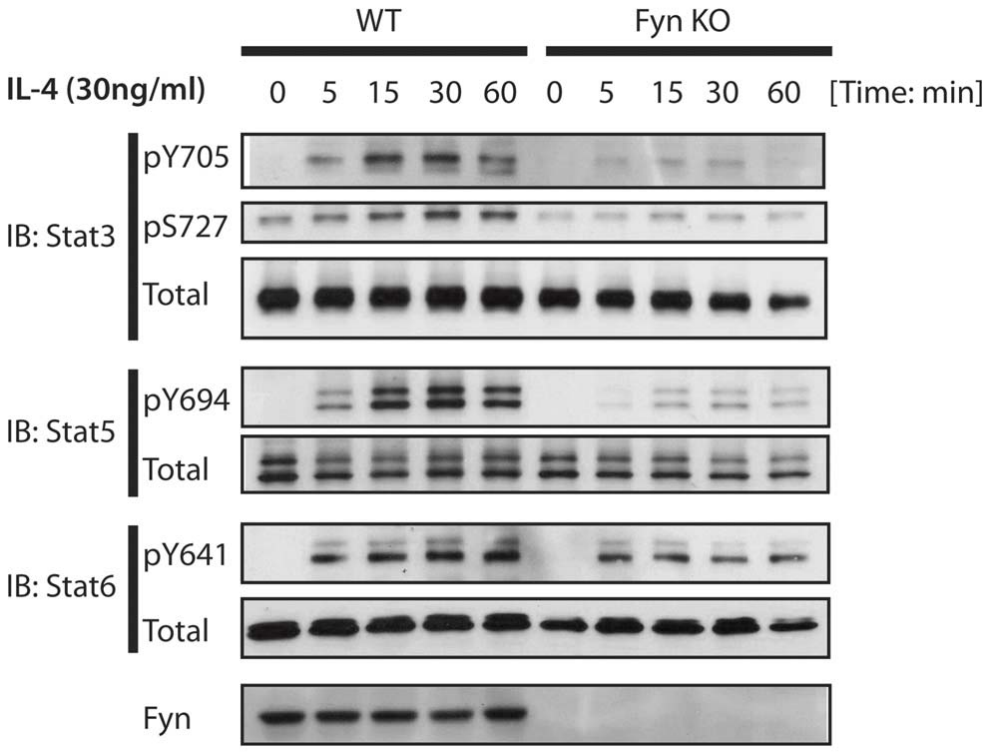
Fyn KO B cells have impaired STAT3 and STAT5 activation upon IL-4 stimulation. WT and Fyn KO naive B cells were isolated from mice and stimulated with IL-4 (30 ng/ml) at 37°C for indicated times. Cells were lysed and phosphorylated forms of STAT3 (pY705 and pS727), STAT5 (pY694) and STAT6 (pY641) were assessed by western blot. Non-phosphorylated proteins were used as loading controls. Representative image of 3 independent experiments.

The IL-4 receptor plays an important role in B cell survival and isotype switch to IgG1 and IgE. Previous studies reported that Fyn KO T cells have altered IL-4 secretion [28], [32], and that Fyn KO B cells had deficient IL-5 responses [18]. Therefore, we hypothesized that B cell IL-4 receptor signaling might be affected by the absence of Fyn kinase. To test this hypothesis, we stimulated WT and Fyn KO naïve B cells with 30 ng/ml of IL-4 for a total of 60 minutes distributed over five time points. While STAT6 phosphorylation seemed unaffected by Fyn deficiency, STAT3 and STAT5 phosphorylation were significantly impaired (Figure 6). Total expression of STAT3, STAT5 and STAT6 was unaffected by the loss of Fyn. Interestingly, altered IL-4R expression did not explain the inability to activate STAT3 and STAT5, since IL-4R expression was similar among WT and Fyn KO cells (data not shown). These findings demonstrate the role of Fyn kinase downstream of IL-4R signaling in B cells and delineate the contribution of this kinase in STATs activation.

## Discussion

Fyn-deficient mice have been studied extensively. In fact, previously published reports have demonstrated that Fyn kinase is not only required for NKT cell development [33] but is also involved in T cell activation and cytokine production [29], [32], [34]. Some studies have demonstrated that Fyn is important for antibody production *in vivo,* although others failed to identify such a role [30], [34], [35]. We noted that Fyn KO mice have delayed kinetics in the production of antigen-specific IgG1 and IgG2c. Moreover, these mice had fewer numbers of GC B cells following immunization, similar to those of IL-4 KO mice. Indeed, IL-4-deficient mice have smaller GCs than WT mice [36]. Given the fact that Fyn is important for IL-4 production, it is possible that reduced IL-4 leads to fewer GC B cells. Along with defective GC formation, we noticed a decrease in splenic PCs and T_FH_. Previous work demonstrated that SAP-Fyn interactions were dispensable for T_FH_ development, as T_FH_ cells developed normally in SAP^R78A^ mice [19]. Therefore, our results suggest that Fyn regulates T_FH_ differentiation in a SAP-independent manner.

Given that Fyn is involved in cytokine production, it is difficult to delineate whether the defects in immunoglobulin production observed in vivo result from B cell-intrinsic defects or to altered T cell cytokine production. However, the fact that Fyn-deficient B cells exhibited impaired antibody production *in vitro* shows that Fyn can influence B cell activation. Interestingly, Fyn-deficient B cells proliferated normally, suggesting a role for Fyn in antibody class switching. While our *in vitro* studies demonstrated a B cell-intrinsic defect, this may not be the full explanation *in vivo*. It is likely that both B cell- and T cell-expressed Fyn play a role in humoral responses.

We show that Fyn KO mice have significantly reduced basal IgE, IgG1 and IgG2c levels. Following immunization, these mice had altered kinetics regarding NP-specific IgG1 and IgG2b production, and a dramatic defect in NP-specific IgG2c secretion. These defects correlated with reduced GC B cells, T_FH_ cells and splenic PCs. Our results thus demonstrate that Fyn-mediated signaling is necessary for an optimal humoral response, especially in regards to IgG2c production. We show that B cell IL-4 receptor stimulation triggers STAT3 and STAT5 phosphorylation as previously reported [20], [37]. Furthermore, we observe that STAT3 and STAT5 phosphorylation downstream of the IL-4 receptor is Fyn-dependent, as Fyn-KO B cells displayed significantly impaired phosphorylation. In addition to a role for Fyn kinase in IL-4-mediated B cell and T cell activation [38], [39], we recently showed that Fyn is required for IgE-induced STAT5 activation in mast cells [40]. Our results thus demonstrate that IL-4 signaling requires Fyn to activate STAT3 and STAT5, and that this may partly explain the impaired CSR in Fyn KO B cells.

Although previous reports suggested that IL-4 mediated phosphorylation of STATs required IL-4Rα, IL-13Rα and the common gamma chain (γc), some others showed that this mechanism might be only partially dependent of Janus kinases (JAKs), implying that other kinases could be required to accomplish complete STAT activation [37], [41]. Further studies demonstrated that STAT5 is critical for B cell survival, development and immunoglobulin gene rearrangements in pro-B cells [42], [43]. Consequently, we realize that it is crucial to dissect the signaling circuitry underlying STAT activation in order to understand their impact in homeostasis and pathologies. For example, studies showed that STAT dysregulation contributes to lymphoma etiology [44]. In fact, while constitutive phosphorylation of STAT6 is known to be a distinctive feature of classical Hodgkin’s lymphoma, constitutive STAT3 activation is commonly encountered in both Hodgkin’s and non-Hodgkin’s lymphoma patients [44]. Adding to these findings, we show that IL-4-mediated STAT3 and STAT5 activation is Fyn-dependent in B cells, and that Fyn deletion impairs antibody production following immunization. Consistent with our findings, it has been demonstrated that STAT3 regulates plasma cell differentiation [45].

The role of Fyn in GC formation was assessed by Cannons, et al. They compared immunized mice from the C57B6, and 129 backgrounds to Fyn KO mice on a mixed C57B6.129 background. In these experiments, C57B6 mice had fewer GC B cells than 129 mice, while Fyn KO mice had GC B cell numbers comparable to C57/B6 WT mice. The authors concluded that Fyn was dispensable for GC formation [19]. However, the variable genetic backgrounds may have masked an effect. We find that among mice of the same age and background, Fyn deficiency reduces the germinal center population in a manner consistent with the observed reduction in antibody production. As previously mentioned, it has been reported that humoral responses to T-dependent antigens are normal among Fyn KO mice 7 or 30 days post-immunization [18], [19]. In these studies, however, antigen-specific IgG2c was not measured. Measurement of total antigen-specific IgG might have masked the defect in antibody production we report here.

Davidsons et al. demonstrated that C57B6 Fyn-deficient mice had relatively normal levels of OVA-specific IgG1 and impaired antigen-specific IgE production following ova-alum immunization [46]. These results are consistent with our finding that C57B6x129sv have markedly diminished basal IgE levels and normal amounts of NP-specific IgG1 levels.

The data regarding the role of Fyn in cytokine production and Th2 cell differentiation is somewhat conflicting. While some have reported that Fyn KO mice have impaired IL-4 production [28], others demonstrated that Fyn KO T cells preferentially differentiate into Th2 cells [29], [35]. Consistent with these findings, Fyn KO mice showed exacerbated responses in a mouse model of allergic airway inflammation [32]. In fact, it is possible that Fyn is involved in the secretion of both IL-4 and IFN-γ. These cytokines promote the production of different Ig isotypes, so the question of which cytokine is more impaired might depend on the physiological context. While IL-4 promotes IgG1 and IgE class switching, IFN-γ induces IgG2a production [47]–[49]. Of note, IgG2c is thought to be controlled in a similar fashion to IgG2a. Here we demonstrate that Fyn-KO mice have dramatically reduced basal and antigen-specific IgG2c levels, consistent with IFN-γ production being impaired in Fyn-KO mice. In addition to contributing to the regulation of IL-4 in T cells [1], [19], [28], [43], [47], [50], our results suggest that Fyn also regulates antibody production in a B cell-intrinsic manner, as Fyn-deficient B cells showed defective antibody production following *in vitro* stimulation.

IgG subclasses bind to specific IgG receptor family members: FcγRI, FcγRIII and FcγRIV are activating receptors, while FcγRIIB is inhibitory. In fact, recent studies conducted in mice show that antigen-specific IgG1 preferentially binds to the inhibitory IgG receptor FcγRIIB with the highest affinity [51]. The ligation of IgG1 to FcγRIIB leads to increased phosphatase mobilization through ITIM activation, suppressing cell activation. Furthermore, the same study provided evidence that IgG2a binds with moderate and high affinity to the activating IgG receptors FcγRIII and FγRIV, respectively. Therefore, IgG2a is capable of eliciting cell activation upon receptor ligation. In terms of physiological relevance, high titers of antigen-specific IgG1 may be inhibitory, while low IgG2a titers might enhance susceptibility to infection. Therefore, understanding how Fyn kinase specifically regulates the production of IgG2a can contribute to vaccine design.

The results presented here further our knowledge of B cell biology by identifying a role for Fyn kinase in B cell IL-4 receptor signaling and subsequent STAT3 and STAT5 activation. Furthermore, we show that Fyn kinase selectively regulates immunoglobulin subclass production, consequently affecting the optimal humoral response.
